# Assessing the feasibility of a pre-triage photo and questionnaire protocol in GP triage: a quality improvement study

**DOI:** 10.1017/S1463423626101169

**Published:** 2026-04-17

**Authors:** Karan Gupta, Caroline Thurston, Harvey McGarry, Angela Bennett

**Affiliations:** University of Cambridge School of Clinical Medicine, UK

**Keywords:** efficiency, general practice, patient management, photo requests, quality improvement, triage

## Abstract

**Background:**

Efficient triage in general practice is critical to optimize appointment allocation and minimize patient delays. Delays in receiving clinical information, such as photographs or symptom questionnaires, lead to unnecessary consultations and inefficiencies. This study evaluated the feasibility and impact of a structured pre-triage protocol requesting photos and questionnaires for common conditions (skin, eye, tonsillitis, and urinary tract infections).

**Methods:**

A pre-post intervention quality improvement project was conducted in a UK general practice. Triage administrators were instructed to proactively request photographs for skin and eye complaints and symptom questionnaires for tonsillitis and UTIs at initial patient contact. Outcomes included process metrics (number of pre-triage requests, proportion of cases managed directly by the triage GP) and subjective measures of ease, speed, satisfaction, and confidence.

**Results:**

The protocol increased photo requests for skin (mean increase 4.0/session, Cohen’s *d* = 7.77) and eye (2.2/session, *d* = 4.09) conditions, while questionnaire requests remained unchanged. The proportion of skin cases managed directly by the triage GP increased significantly (from 0.2 to 2.2 cases/session, *d* = 1.65), and eye case management also improved. Questionnaire-based pathways showed minimal change in efficiency or direct management. Subjective feedback indicated a slight reduction in triage speed, but ease and satisfaction were maintained, while diagnostic confidence increased, particularly for photo-supported conditions.

**Conclusion:**

A structured pre-triage protocol is feasible, acceptable, and potentially effective in enhancing triage efficiency, particularly for visually assessable conditions like skin and eye presentations. By enabling earlier access to essential information, such protocols may reduce unnecessary consultations, improve workflow, and support clinician confidence.

## Background

General practice appointments are a valuable and limited resource, where medical management plans are developed and referrals are made. Backlogs in primary care waiting lists lead to disease progression and increased complications before patients can attend these appointments, ultimately limiting the effectiveness of interventions. Therefore, minimizing patient delays and optimizing triage efficiency is imperative.

Triage sessions in general practice were introduced to prioritize patients and optimize appointment allocation. One notable development is the implementation of a triage GP at a Swedish primary health centre in November 2015, bringing medical input alongside administrative support to prioritize patients (Gelin *et al*., 2023). Triage aims to allocate patients to the appropriate level of care – urgent same-day appointments, less urgent reviews, or routine consultations – and assess whether consultations can be remote or require face-to-face evaluation. Delays frequently occur when additional information, such as clinical photographs or symptom questionnaires, is required for remote management. High workload pressures often lead triage doctors to proceed with consultations without waiting for patient responses, reducing appointment availability, increasing waiting times, and contributing to inefficiencies.

There is significant potential to enhance the triage model by integrating pre-triage protocols that proactively request relevant clinical information from patients. In English GP practices, while patients may upload photos or complete questionnaires, this is not consistently done, and there is limited evidence on the feasibility and impact of structured pre-triage interventions. In our practice, pre-triage questionnaires indicated some benefit, but uncertainty remained regarding which conditions benefit most from pre-triage input, and whether photos or questionnaires are more effective.

Evidence from dermatology supports the value of photo triage in general practice. For example, the Dermatology Photo Triage referral pathway, piloted in 2018 and now adopted by 96% of GP practices in Northern Ireland, allows high-quality photographs to be sent to specialists for rapid diagnosis and management, bypassing hospital visits (Department of Health, 2023). Between March and December 2022, over 3,300 referrals were made, primarily for suspected skin cancer, with rapid specialist response (Department of Health, 2023). BMA guidance recommends using photos for conditions like skin infections (BMA, 2023a; BMA, 2023b), and NHS England highlights the integration of digital tools, including photo submissions, to improve triage efficiency (NHS England, 2020).

**Figure 1.**
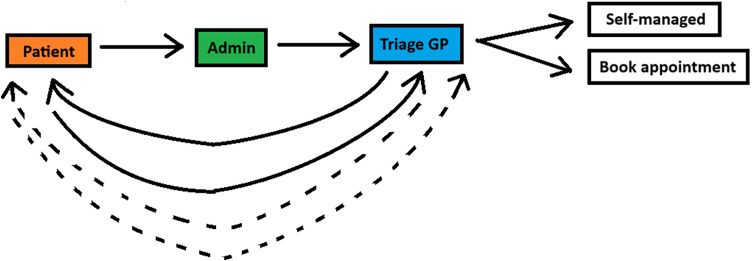
Schematic diagram of the previous triage pathway, highlighting the iterative back-and-forth exchanges between GPs and patients due to delayed information requests, leading to inefficiencies and unnecessary GP consultations.

In the existing triage system at our practice (Figure 1), patients initiate contact via an electronic message, which is assessed by triage administrators before being placed in the GP’s triage queue. Patients may also call the practice, in which case triage staff collect relevant information and enter it into the electronic system for the triage GP. While patients could upload photos proactively, GPs predominantly request additional information such as photos or questionnaires, leading to iterative back-and-forth communication between the GP and patient, repeated triage cycles, and unnecessary delays. This workflow reflects common practice in English GP surgeries using digital-first triage systems and underscores the need for structured pre-triage protocols to improve efficiency and reduce unnecessary consultations.

## Methods

This study was conducted as a pre-post intervention quality improvement project (QIP) in a single GP practice in England. The primary aim was to assess the feasibility of a pre-triage photo and questionnaire protocol, focusing on workflow integration, acceptability to triage administrators and GPs, and preliminary indications of impact on patient management. The study was structured around the placement of penultimate-year medical students, who attend the practice in two-week blocks. One block was used for planning the QIP, and the subsequent block involved pre-intervention, intervention, and post-intervention phases conducted over 10 working days. This timeframe was chosen because it aligned with student placement schedules and allowed sufficient time to assess feasibility and staff engagement, while acknowledging that it was not long enough to draw conclusions regarding sustained efficiency or long-term outcomes.

**Figure 2.**
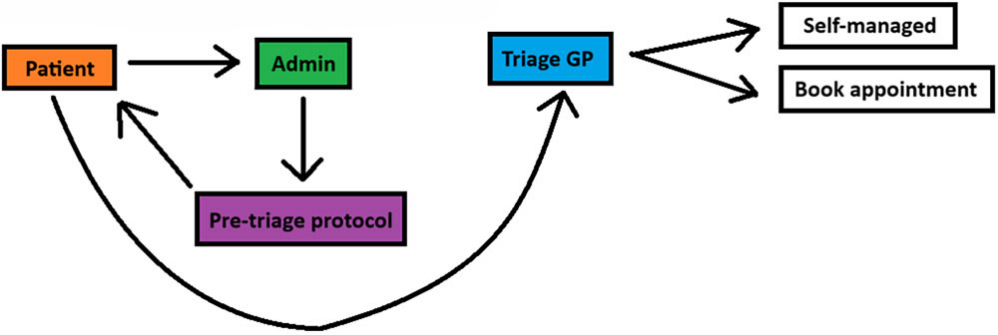
Schematic diagram of the new triage pathway, demonstrating the integration of proactive pre-triage data collection for common conditions, enabling faster clinical decision-making and reducing the need for additional GP appointments.

The intervention involved a structured pre-triage protocol (Supplementary File 1) targeting the four most common conditions managed in triage: skin problems, eye problems (conjunctivitis), tonsillitis, and urinary tract infections (UTIs). Triage administrators were instructed to proactively request additional information at the first patient encounter via Accurex messages: photo submissions for skin and eye conditions, and symptom questionnaires for tonsillitis and UTIs. These requests were immediately added to the GP’s folder without waiting for patient responses. The protocol was designed to integrate seamlessly into routine triage workflow while maintaining patient safety (Figure 2). Triage administrators received structured teaching on the protocol, including symptom recognition, message criteria, and escalation procedures. A printed reference guide was displayed at the triage workstation to support adherence, and medical students rotated alongside administrators to provide real-time guidance and ensure consistency.

Primary outcomes focused on feasibility, assessed through subjective measures such as ease, speed, satisfaction and perceived diagnostic accuracy reported by triage administrators (Supplementary File 2) and GPs (Supplementary File 3) in managing cases with pre-triage information. Confidence in managing patients directly within triage, rather than referring them for a further appointment, was assessed separately for GPs (Supplementary File 4). Subjective measures were collected via anonymous questionnaires sent to 10 triage GPs and 5 administrators, which were piloted informally for clarity and relevance.

Secondary outcomes captured process metrics, including the number of pre-triage requests dispatched per session, the number of cases the triage GP was awaiting a patient response from, and the proportion of patients managed directly by the triage GP without requiring further consultation. These outcomes assess workflow integration and preliminary impact on case management. Data were collected using two tally charts: one completed by triage administrators to record pre-triage requests (Supplementary File 5), and one by the triage GP to record case management decisions (Supplementary File 6). Administrators and GPs were trained in a joint teaching session on consistent completion of the tally charts to ensure data reliability.

As this was a feasibility study, no formal a priori power calculation was undertaken. Given the small sample size, analyses were exploratory and not designed to detect statistically significant differences. Descriptive statistics, including means and standard deviations, are therefore presented to characterize observed trends. As no directional hypotheses were specified, two-tailed unpaired t-tests were used to compare pre- and post-intervention measures, with effect sizes reported to aid interpretation. These inferential results are presented for contextual purposes only and interpreted cautiously. All analyses were conducted using standard spreadsheet software.

## Results

In this GP practice, a single triage doctor and triage administrator handle the morning triage, with the number of patients varying between 25 to 50 per session. Over the course of the 10-day project, the average number of patients triaged per session was 42. Among these, skin-related issues accounted for approximately 8 patients, eye-related problems for 4, sore throats for 5, and urinary problems for 6 patients on average.

Following implementation of the pre-triage protocol, triage administrators consistently incorporated photo requests for skin and eye presentations into routine workflow (Table 1). The magnitude of change was large for both conditions, with skin photo requests increasing by an average of 4.0 per session (from 0.8 to 4.8; Cohen’s *d* = 7.77) and eye photo requests increasing by 2.2 per session (from 0.6 to 2.8; Cohen’s *d* = 4.09). These large effect sizes indicate a substantial and practically meaningful shift in administrator behaviour following introduction of the protocol. In contrast, questionnaire requests for tonsillitis and UTIs remained stable at approximately four requests per session, with negligible effect sizes (Cohen’s *d* ≤ 0.27).

**Table 1.** Pre- and post-intervention mean (±SD) number of pre-triage requests per session for targeted conditions, with exploratory *t*-test results, *p*-values, and effect sizes (Cohen’s *d*) following implementation of the pre-triage protocol

	Admin requests					
	Pre-intervention	Post-intervention	Difference	*t*-test	*p*-Value	Cohen’s *d*
Skin	0.8 ± 0.2	4.8 ± 0.7	4.0	−9.428	**<0.001**	**7.77**
Eyes	0.6 ± 0.3	2.8 ± 0.7	2.2	−4.919	**0.001**	**4.09**
Tonsillitis	3.8 ± 0.7	3.8 ± 0.2	0.0	0.000	1.000	0.00
UTIs	3.6 ± 0.8	3.8 ± 0.7	0.2	−0.364	0.724	0.27

The number of cases the triage GP was awaiting a patient response from decreased by 26%, from a mean of 3.4 to 2.5 patients per session (Table 2). Although this represents a moderate reduction in absolute workload (Cohen’s *d* = 0.70), the variability observed suggests that this change should be interpreted as a preliminary signal rather than a definitive efficiency gain. The proportion of patients managed directly by the triage GP increased meaningfully for conditions supported by photos. Direct management of skin-related presentations increased by an average of 2.0 cases per session (from 0.2 to 2.2; Cohen’s *d* = 1.65), while eye-related cases increased by 1.2 per session (from 0.8 to 2.0; Cohen’s *d* = 1.39), indicating large and practically meaningful shifts in case handling. No comparable changes were observed for tonsillitis or UTI cases, where effect sizes were small (Cohen’s *d* ≤ 0.33), suggesting that pre-triage information did not substantially prevent these patients being booked for further consultations.

**Table 2.** Pre- and post-intervention mean (±SD) number of requests from the triage GP awaiting a patient response, number managed directly within triage for each condition, with exploratory *t*-test results, *p*-values, and effect sizes (Cohen’s *d*) following implementation of the pre-triage protocol

	Direct management					
	Pre-intervention	Post-intervention	Difference	*t*-test	*p*-Value	Cohen’s *d*
Waiting frequency	3.4 ± 1.38	2.5 ± 1.17	−26%	1.784	0.912	0.70
Skin	0.2 ± 0.2	2.2 ± 1.7	2.0	−3.244	**0.012**	**1.65**
Eyes	0.8 ± 0.7	2.0 ±1.0	1.2	−2.058	0.074	**1.39**
Tonsillitis	1.0 ± 0.5	1.2 ± 1.2	0.2	−0.343	0.740	0.22
UTIs	1.2 ± 0.7	1.0 ± 0.5	−0.2	0.408	0.694	0.33

Subjective feedback from triage administrators demonstrated minimal overall change following implementation of the pre-triage protocol (Table 3). Perceived ease and satisfaction increased modestly by approximately 5%, while perceived speed decreased by a similar margin. The small magnitude of these changes, together with overlapping variability between pre- and post-intervention ratings, suggests that the protocol introduced a slight increase in administrative input via proactive information gathering without materially affecting workflow efficiency, staff acceptability or staff experience.

**Table 3.** Pre- and post-intervention mean (±SD) subjective ratings (scale 1–10) of ease, speed, and satisfaction reported by triage administrators, with exploratory *t*-test results and p-values following implementation of the pre-triage protocol

	Admin ratings (scale 1–10)				
	Pre-intervention	Post-intervention	Difference (%)	*t*-test	*p*-Value
Easiness	7.6 ± 1.3	8.0 ± 0.5	5	−0.667	0.524
Speed	8.4 ± 1.3	8.0 ± 1.5	−5	0.535	0.608
Satisfaction	8.4 ± 0.8	8.8 ± 1.7	5	−0.566	0.587

Conversely, subjective feedback from triage GPs indicated a modest shift in perceived experience following implementation of the pre-triage protocol (Table 4). Ratings of ease, speed, and satisfaction decreased by 6–10%, while perceived diagnostic accuracy remained stable. The reduction in perceived speed suggests that managing cases with more complete pre-triage information may require additional cognitive or decision-making time during triage sessions. However, the relatively small magnitude of change across all domains, alongside stable accuracy ratings, indicates that the protocol did not adversely affect clinicians’ confidence in their clinical decision-making. Overall, these findings suggest that while the intervention may have slowed the pace of triage work for GPs, it remained acceptable and clinically manageable within routine practice.

**Table 4.** Pre- and post-intervention mean (±SD) subjective ratings (scale 1–10) of ease, speed, accuracy, and satisfaction reported by triage GPs, with exploratory *t*-test results and *p*-values following implementation of the pre-triage protocol

	GP ratings (scale 1–10)				
	Pre-intervention	Post-intervention	Difference (%)	*t*-test	*p*-Value
Easiness	7.9 ± 1.43	7.3 ± 0.9	−8	1.242	0.230
Speed	9.0 ± 0.67	8.1 ± 0.99	−10	2.212	**0.040**
Accuracy	6.8 ± 0.84	6.7 ± 0.9	−1	0.239	0.813
Satisfaction	8.5 ± 1.17	8.0 ± 0.67	−6	1.168	0.258

Confidence ratings demonstrated a notable shift in triage GPs’ perceived ability to manage different condition groups following implementation of the pre-triage protocol (Table 5). Prior to the intervention, confidence in managing skin and eye presentations was lower (mean 4.8/10) than for tonsillitis and UTI cases (mean 6.2/10), representing a difference of approximately 30%. After implementation, this disparity was no longer evident, with confidence ratings converging at around 6/10 across all conditions. This was primarily driven by increased confidence in managing skin and eye presentations, where photo-based information was routinely available, while confidence for tonsillitis and UTI cases remained broadly stable.

**Table 5.** Pre- and post-intervention mean (±SD) confidence ratings (scale 1–10) reported by triage GPs for managing patients with photo-based and questionnaire-based pre-triage information, with exploratory *t*-test results and *p*-values following implementation of the pre-triage protocol

	GP confidence rating 1–10				
	Pre-intervention	Post-intervention	Difference (%)	*t*-test	*p*-Value
Skin	4.7 ± 1.34	5.9 ± 1.43	26	−2.278	**0.035**
Eyes	4.9 ± 0.54	6.1 ± 2.32	24	−2.241	**0.038**
Tonsillitis	6.0 ± 1.11	5.8 ± 1.73	−3	0.375	0.712
UTIs	6.4 ± 1.38	6.1 ± 1.88	5	0.526	0.605

## Discussion

These findings demonstrate a feasible and practice-acceptable shift in how pre-triage information was requested and used following implementation of an administrative protocol. Prior to the intervention, this practice had already experimented with pre-triage questionnaires, resulting in relatively high baseline questionnaire use and comparatively fewer photo requests. Post-intervention, photo requests for skin and eye presentations increased to levels comparable with questionnaires (Table 1), suggesting that questionnaire use was already near optimal, whereas photo-based triage had previously been underutilized. In practices where neither questionnaires nor photo requests are currently embedded, similar protocols may support adoption of both modalities; however, the magnitude of downstream impact on direct triage management may differ. In settings where one modality is already well established, incremental gains are likely to be more modest, as observed here.

The reduction in the number of patients the triage GP was awaiting responses from (Table 2), although not statistically significant, suggests a potentially meaningful improvement in workflow efficiency. From a feasibility standpoint, even small reductions may translate into smoother triage flow and fewer delays, particularly in high-volume practices. While clinicians remain active while awaiting responses, reducing reliance on follow-up information may decrease the likelihood of pre-emptively booking unnecessary appointments. This, in turn, could help reduce waiting lists and overall waiting times for patients associated with the practice.

In terms of patient management, the intervention was associated with a marked increase in the proportion of skin and eye cases managed directly within triage. Notably, post-intervention management of photo-supported conditions exceeded that of questionnaire-supported conditions such as tonsillitis and UTIs (Table 2). The increase in skin condition management reached statistical significance, while eye condition management increased substantially but did not meet conventional thresholds for significance. These findings should be interpreted cautiously given the exploratory nature of the study, but they nonetheless suggest that photo-based information may be particularly effective in supporting triage-level resolution of certain presentations.

An important distinction emerged between photo requests and questionnaire requests. Although their overall frequencies became similar post-intervention (Table 1), questionnaires were far less likely to result in direct triage management (Table 2). In absolute terms, skin and eye cases managed directly were almost double those of tonsillitis and UTIs. This supports the feasibility of prioritizing photo-based triage for conditions where visual assessment is central and aligns with broader primary care experience that clinicians often place greater trust in visual data than in symptom checklists alone. Questionnaires rely heavily on patient interpretation, health literacy, and subjective symptom reporting, which may introduce uncertainty and limit decisional confidence.

The difference between skin and eye conditions is also noteworthy. Although eye photo requests increased significantly, this did not translate into an equally large increase in direct management. One plausible explanation relates to clinical risk: skin conditions typically evolve over longer timeframes and often have simpler management pathways, whereas eye conditions may deteriorate rapidly and carry a higher risk of serious complications. This may also explain why confidence in managing eye conditions increased significantly (Table 5), while clinicians remained appropriately cautious about resolving them definitively within triage.

Effect sizes (Cohen’s *d*) were used to provide context for observed changes, given the exploratory nature and limited sample size of this feasibility study. Large effect sizes for photo-supported conditions (skin and eye presentations) suggest practically meaningful shifts in triage behaviour, even where statistical significance was not consistently observed. In contrast, small effect sizes for questionnaire-supported conditions (tonsillitis and UTIs) indicate minimal practical change following the intervention, supporting the interpretation that these pathways were already functioning near capacity. Moderate effect sizes observed for some workflow measures (e.g. requests awaiting patient response) may reflect early efficiency signals that warrant further investigation in larger, adequately powered studies rather than definitive conclusions. Overall, Cohen’s *d* values were interpreted as indicators of feasibility and magnitude of change rather than effectiveness, helping to distinguish between trivial, moderate, and practically meaningful impacts of the intervention.

Subjective feedback provides further insight into feasibility. Triage GPs reported no meaningful change in perceived ease or satisfaction but did report a significant reduction in perceived speed (Table 4), likely reflecting the additional cognitive and decision-making steps required when actively managing cases within triage rather than deferring them. Importantly, perceived diagnostic accuracy remained unchanged. Confidence increased significantly for skin and eye presentations, reinforcing the practical value of photos in supporting decision-making (Table 5). Although triage GPs experienced triage as slower, this does not necessarily indicate reduced system efficiency. From a practice-level perspective, the intervention may shift workload earlier in the pathway, reducing the number of downstream appointments and repeat contacts. In contrast, triage administrators report no significant changes in ease, speed, or satisfaction (Table 3), suggesting the protocol is acceptable and manageable within existing administrative roles despite slightly increased involvement.

Qualitative feedback highlighted important contextual considerations. Clinicians noted that confidence varies with case complexity, indicating that these results reflect typical rather than extreme presentations. It was also emphasized that waiting for patient responses does not equate to idle time, as clinicians multitask; however, earlier access to higher-quality information may still reduce premature decision-making and unnecessary escalation. Additionally, a triage administrator noted that triage speed is more dependent on the GP than the admin team. However, if this is the case, then implementing this protocol for administrators may still be justified as a way to enhance the efficiency of triage doctors. Both GPs and administrators identified practical refinements that could further enhance efficiency, including administrative verification of patient identity and contact details, documenting prior consultations or investigations, and highlighting medication allergies. Administrators also identified delays in patient responses – particularly outside triage hours – as a challenge, suggesting routing responses directly to GP folders can improve continuity and oversight.

Generalisability of these findings should be considered in light of the organizational context. This study was conducted in a single UK general practice using a triage-GP model supported by digital messaging. Primary care triage systems vary widely internationally and even within the UK, ranging from practices without GP-led triage, to those with GP triage but minimal administrative protocols. In systems without a dedicated triage GP, the direct management benefits observed here may be less transferable. Conversely, in practices already using GP triage but lacking structured administrative protocols, similar interventions may be particularly applicable. The feasibility demonstrated here is likely most relevant to practices with some degree of digital infrastructure and clinician involvement in triage. However, the underlying principle – aligning information requests with clinical decision-making needs – may be transferable across systems, albeit requiring local adaptation.

This study has several limitations. Data were drawn from a single practice with a small number of clinicians and administrators, limiting representativeness. The exploratory design and absence of a priori power calculations mean that findings should be interpreted as signals of feasibility rather than definitive evidence of effectiveness. Subjective measures are susceptible to response bias, and short-term follow-up may not capture longer-term adaptation or efficiency gains. Additionally, the intervention was only tested on the four most common conditions in triage; applicability to other conditions requires further study. Finally, patient outcomes were not assessed, and future work should examine whether earlier resolution translates into improved patient satisfaction or reduced healthcare utilization.

## Conclusion

This QIP demonstrates that a structured pre-triage photo and questionnaire protocol is feasible and acceptable within a general practice triage system, particularly for skin and eye presentations. The observed patterns suggest that incorporating targeted photo requests can support triage decision-making and increase the proportion of cases managed directly within triage, without adversely affecting administrative acceptability. While these findings indicate potential efficiency gains, the short duration and small scale of the study mean that conclusions should be interpreted as preliminary signals of feasibility rather than definitive improvements in efficiency.

Reductions in onward booking for selected conditions suggest that, if sustained, this approach could meaningfully contribute to appointment availability and patient experience. For example, the observed increase in direct management of skin and eye conditions corresponds to several potential appointments avoided per triage session. Theoretically, 3 appointment bookings saved per triage session corresponds to 15 per week and 624 per year, a substantial number. However, these projections remain theoretical and require confirmation through longer-term implementation and more robust outcome measurement. Importantly, the additional responsibilities placed on administrative staff appeared manageable, with no deterioration in subjective experience, supporting the practical viability of the protocol.

Future work should involve longer implementation cycles, ideally across multiple practices, to evaluate sustainability, scalability, and contextual variation. Mixed-methods evaluation incorporating patient perspectives, compliance with information requests, and condition-specific response patterns would strengthen understanding of how and why the intervention works. Quantitative metrics such as the proportion of eligible patients receiving pre-triage requests, rather than session-based tallies, would further improve evaluation accuracy. Integration of protocols directly into electronic triage systems and expansion to additional conditions may also enhance impact.

On the whole, this study gives some confidence to general practices who want to explore novel ways to increase efficiency in primary care triage. By taking the Swedish model, and attempting to improve it by incorporating elements of the Dermatology Photo Triage referral pathway from Northern Ireland, we have developed a seemingly successful pre-triage protocol at our practice in England. Fascinatingly, the fact that skin conditions seem the best to triage in both our study and the Northern Ireland system may suggest dermatology is an ideal specialty to align more closely with primary care. Perhaps in future, general practices in Europe will be able to send skin photos from triage sessions internationally directly to dermatology services with shorter waiting lists for their clinical opinion, saving time and appointments and relieving pressure on primary care.

## Supporting information

10.1017/S1463423626101169.sm001 Gupta et al. supplementary material 1 Gupta et al. supplementary material

10.1017/S1463423626101169.sm002 Gupta et al. supplementary material 2 Gupta et al. supplementary material

10.1017/S1463423626101169.sm003 Gupta et al. supplementary material 3 Gupta et al. supplementary material

10.1017/S1463423626101169.sm004 Gupta et al. supplementary material 4 Gupta et al. supplementary material

10.1017/S1463423626101169.sm005 Gupta et al. supplementary material 5 Gupta et al. supplementary material

10.1017/S1463423626101169.sm006 Gupta et al. supplementary material 6 Gupta et al. supplementary material

## Data Availability

https://doi.org/10.17863/CAM.116453

## Supplementary material

The supplementary material for this article can be found at https://doi.org/10.1017/S1463423626101169.
