# Supplementary material for: Assessing the feasibility of a pre-triage photo and questionnaire protocol in GP triage: a quality improvement study
Source: Prim Health Care Res Dev. 2026 Apr 17;27:e49. doi: 10.1017/S1463423626101169 (PMC13125267; doi:10.1017/S1463423626101169)
Supplement: Gupta et al. supplementary material 1 — Gupta et al. supplementary material [file S1463423626101169sup001.docx]

Triage Administrator Proposed Protocol

1. Skin, eyes, throat conditions:

If the patient is complaining of any of these 3 conditions, do they mention any of the following symptoms: redness, swelling, discharge, itchiness, soreness?

If yes, send the following Accurex message to the patient and add the Accurex to the doctor’s folder (do not wait for response):

- Skin condition patient triage request
- Eye condition patient triage request
- Sore throat questionnaire

If no, please send directly to the triage doctor’s list.

1. Urination problems

If the patient is complaining of urinary symptoms, do they mention any of the following: painful urination, frequency, urgency, cloudiness, smelliness?

If yes, then send the “UTI questionnaire” and add the Accurex to the doctor’s folder (do not wait for response).

If no, please send directly to the triage doctor’s list.
