# Supplementary material for: Assessing the feasibility of a pre-triage photo and questionnaire protocol in GP triage: a quality improvement study
Source: Prim Health Care Res Dev. 2026 Apr 17;27:e49. doi: 10.1017/S1463423626101169 (PMC13125267; doi:10.1017/S1463423626101169)
Supplement: Gupta et al. supplementary material 2 — Gupta et al. supplementary material [file S1463423626101169sup002.docx]

Triage Administrator Satisfaction Questionnaire

A doctor’s triage list contains many patients, some suggesting simple problems which could be dealt with by the triaging doctor themselves, such as:

- Skin problems
- Conjunctivitis
- Tonsillitis
- Urinary Tract Infection

Doctors have the option to treat/advise the patient after asking them for more information but there may not be enough time to do so if triage is very busy. For this reason, we are proposing a protocol where the triage administrators request photos or send the appropriate questionnaire to patients at the same time as sending the patient’s Accurex message to the triage doctor’s list. We hope to save the doctors a lot of time without costing the admin team much time.

1. Ease of Triage: On a scale of 1-10, how easy do you find the triage process?

Very difficult 1 2 3 4 5 6 7 8 9 10 Very easy

1. Speed of Triage: On a scale of 1-10, how fast do you find the triage process?

Very slow 1 2 3 4 5 6 7 8 9 10 Very fast

1. Overall Satisfaction: What is your overall satisfaction with the triaging process in terms of efficiency and effectiveness on a scale of 1-10?

Very dissatisfied 1 2 3 4 5 6 7 8 9 10 Very satisfied

1. Additional Information: Are there any other pieces of information that you believe could be obtained pre-triage to enhance the efficiency of the process?
