# Supplementary material for: Assessing the feasibility of a pre-triage photo and questionnaire protocol in GP triage: a quality improvement study
Source: Prim Health Care Res Dev. 2026 Apr 17;27:e49. doi: 10.1017/S1463423626101169 (PMC13125267; doi:10.1017/S1463423626101169)
Supplement: Gupta et al. supplementary material 3 — Gupta et al. supplementary material [file S1463423626101169sup003.docx]

Triage GP satisfaction questionnaire

1. Pre-Triage Communication: How often are you waiting for a patient’s response to a message in a triaging session before effectively triaging?

☐ 0

☐ 1

☐ 2

☐ 3

☐ 4

☐ 5+

1. Ease of Triage: On a scale of 1-10, how easy do you find the triage process based on the information provided by the reception staff?

Very difficult 1 2 3 4 5 6 7 8 9 10 Very easy

1. Speed of Triage: On a scale of 1-10, how fast do you find the triage process based on the information supplied by the reception staff?

Very slow 1 2 3 4 5 6 7 8 9 10 Very fast

1. Accuracy of Triage: On a scale of 1-10, how accurate do you find the triage process based on the information supplied by the reception staff?

Very inaccurate 1 2 3 4 5 6 7 8 9 10 Very accurate

1. Overall Satisfaction: What is your overall satisfaction with the clinical triaging process in terms of efficiency and effectiveness on a scale of 1-10?

Very dissatisfied 1 2 3 4 5 6 7 8 9 10 Very satisfied

1. Additional Information: Are there any other pieces of information that you believe could be obtained pre-triage to enhance the efficiency of the process?
