# Supplementary material for: Assessing the feasibility of a pre-triage photo and questionnaire protocol in GP triage: a quality improvement study
Source: Prim Health Care Res Dev. 2026 Apr 17;27:e49. doi: 10.1017/S1463423626101169 (PMC13125267; doi:10.1017/S1463423626101169)
Supplement: Gupta et al. supplementary material 4 — Gupta et al. supplementary material [file S1463423626101169sup004.docx]

Triage GP Information Questionnaire:

A doctor’s triage list contains many patients, some of which suggest simple pathology that could be dealt with by the triaging doctor themselves, such as:

- Skin problems
- Conjunctivitis
- Tonsillitis
- Urinary Tract Infection

Doctors have the option to treat/advise the patient after asking them for more information but there may not be enough time to do so if triage is very busy. For this reason, we are proposing a protocol where the triage administrators request photos or send the appropriate questionnaire to patients before review by the triage doctor.

In response to their Accurex, the admin team will send the following questionnaire. How likely are you to triage to a telephone call with a GP versus prescribing as the triage doctor, for the following:

1. Suspected UTI: “UTI questionnaire”

Most likely to triage as a phone call 1 2 3 4 5 6 7 8 9 10 Most likely to treat/advise myself

1. Skin conditions: “Skin condition patient triage request”

Most likely to triage as a phone call 1 2 3 4 5 6 7 8 9 10 Most likely to treat/advise myself

1. Eye conditions: “Eye condition patient triage request”

Most likely to triage as a phone call 1 2 3 4 5 6 7 8 9 10 Most likely to treat/advise myself

1. Suspected tonsillitis: “Sore throat questionnaire”

Most likely to triage as a phone call 1 2 3 4 5 6 7 8 9 10 Most likely to treat/advise myself
