# Supplementary material for: Assessing the feasibility of a pre-triage photo and questionnaire protocol in GP triage: a quality improvement study
Source: Prim Health Care Res Dev. 2026 Apr 17;27:e49. doi: 10.1017/S1463423626101169 (PMC13125267; doi:10.1017/S1463423626101169)
Supplement: Gupta et al. supplementary material 5 — Gupta et al. supplementary material [file S1463423626101169sup005.docx]

Record of Accurex messages sent

|  | Accurex messages sent (tally chart) | | | | Total |
| --- | --- | --- | --- | --- | --- |
| Date | Throat | UTI | Skin | Eye |  |
| 19/02/2024 |  |  |  |  |  |
| 20/02/2024 |  |  |  |  |  |
| 21/02/2024 |  |  |  |  |  |
| 22/02/2024 |  |  |  |  |  |
| 23/02/2024 |  |  |  |  |  |

Record of Accurex messages sent

|  | Accurex messages sent (tally chart) | | | | Total |
| --- | --- | --- | --- | --- | --- |
| Date | Throat | UTI | Skin | Eye |  |
| 26/02/2024 |  |  |  |  |  |
| 27/02/2024 |  |  |  |  |  |
| 28/02/2024 |  |  |  |  |  |
| 29/02/2024 |  |  |  |  |  |
| 01/03/2024 |  |  |  |  |  |
